# Taiwan’s first clinical reports on the surgical effect of high-frequency deep sclerotomy for treating primary open-angle glaucoma

**DOI:** 10.1186/s12886-025-03881-8

**Published:** 2025-02-20

**Authors:** Wei-Xiang Wang, Mei-Lan Ko

**Affiliations:** 1 School of Medicine, College of Medicine, Taipei Medical University, Taipei, 110 Taiwan; 2 Department of Ophthalmology, National Taiwan University Hospital, Hsin-Chu Branch, Hsin-Chu City, 300 Taiwan; 3 Department of Ophthalmology, College of Medicine, National Taiwan University, Taipei, 110 Taiwan; 4 No. 25, Lane 442, Section 1, Jingguo Rd., North District, Hsinchu City, 300 Taiwan

**Keywords:** Glaucoma, High-frequency deep sclerotomy, Primary open angle glaucoma, Minimally invasive glaucoma surgery

## Abstract

**Background:**

Primary open-angle glaucoma (POAG) leads to elevated intraocular pressure (IOP) and gradual optic nerve damage. In a study by Abushanab et al., high-frequency deep sclerotomy (HFDS) effectively treated patients with POAG. HFDS creates channels through the trabecular meshwork (TM) using a high-frequency electrocautery probe tip, promoting aqueous humor outflow and reducing IOP. In Taiwan, HFDS has been rarely used to treat POAG patients. Therefore, we conducted the first trial and presented the outcomes of two cases to evaluate its effectiveness.

**Case presentation:**

Both patients had long-term primary open-angle glaucoma (POAG) with significant optic nerve damage and visual field loss despite multiple medications. Case 1: A 66-year-old female with a preoperative intraocular pressure (IOP) of 20 mmHg and a history of diabetes mellitus (DM) underwent high-frequency deep sclerotomy (HFDS). Postoperatively, the IOP initially decreased to 12 mmHg without Abstract Pagemedications but reintroduced drops to maintain 13-15 mmHg during follow-up. Case 2: A 50-year-old female with a preoperative IOP of 18 mmHg underwent HFDS. The IOP remained stable between 11 and 13 mmHg postoperatively with a consistent medication regimen.

**Discussion and conclusions:**

HFDS is a minimally invasive glaucoma surgery (MIGS) that effectively lowers the IOP in patients unresponsive to conventional treatments. This report presents two of Taiwan’s first patients with POAG treated by HFDS, showing IOP reductions of 30% and 33.3% over one year with mild corneal endothelial cell loss, which is consistent with previous studies. HFDS demonstrated a significant IOP reduction compared to that in other MIGS techniques and fewer complications than traditional surgeries. Further research should optimize the postoperative management, consider the anatomical differences and pocket healing.

## Key messages

This manuscript presents two case reports on the efficacy of high-frequency deep sclerotomy in treating patients with primary open-angle glaucoma. The findings indicate significant IOP reduction and minimal complications, contributing valuable insights into minimally invasive glaucoma surgery.

## Introduction

The main goal of treatment for primary open-angle glaucoma (POAG), the most prevalent type of glaucoma, is to lower the IOP and control it within a safe range [1]. In a human clinical study performed by Abushanab et al., high-frequency deep sclerotomy (HFDS) successfully achieved effective treatment in patients with POAG [2]. The principle of HFDS is to use a high-frequency electrocautery probe tip to release high-frequency energy, which dissipates the surrounding tissue; hence, this procedure creates channels through the trabecular meshwork (TM), promoting aqueous humor outflow and decreasing IOP. Herein, we describe two of Taiwan’s first patients with POAG treated by HFDS.

## Case presentation

### Case 1

**Fig. 1:**
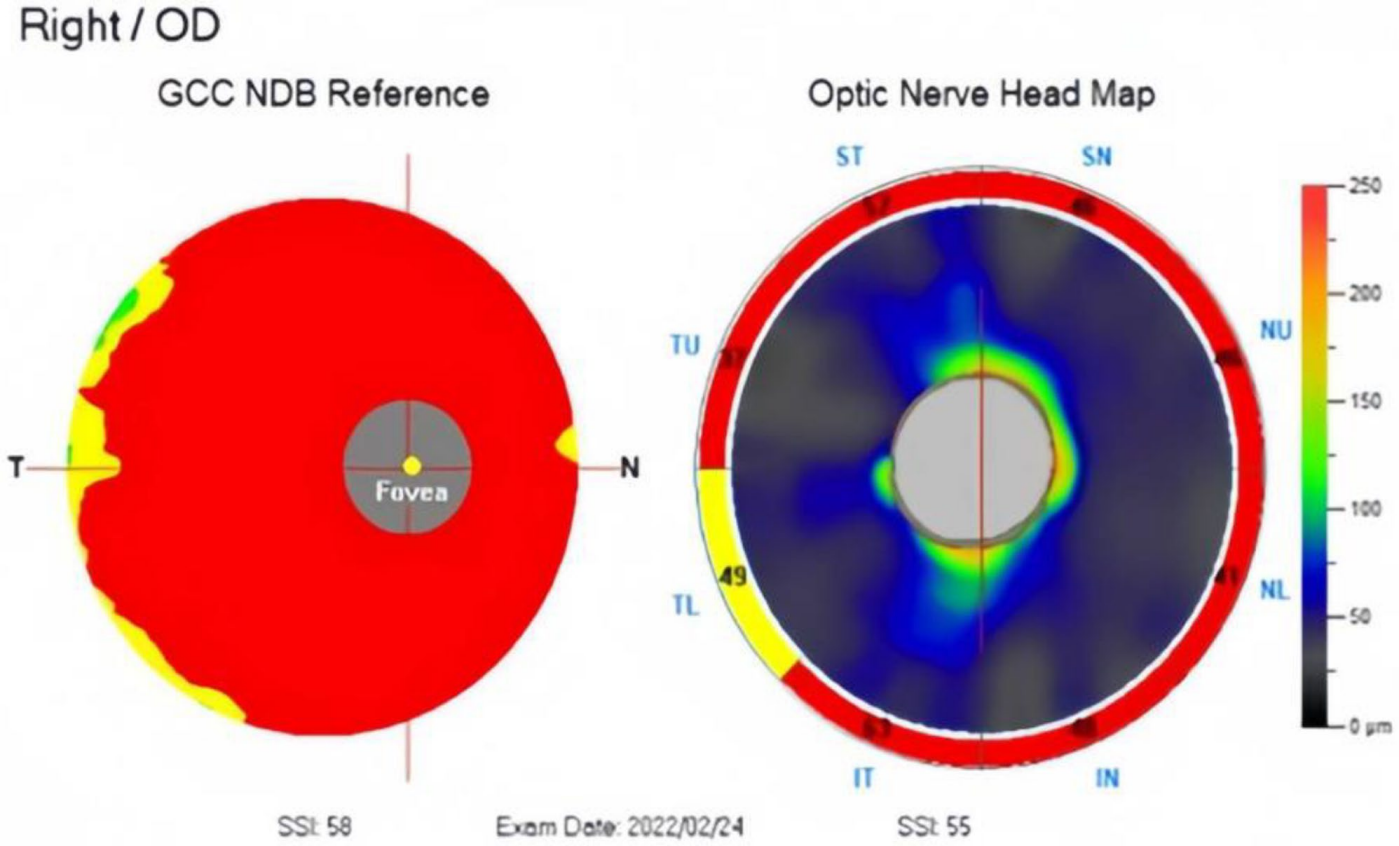
OCT revealed severe thinning in the RNFL and GCC

**Fig. 2:**
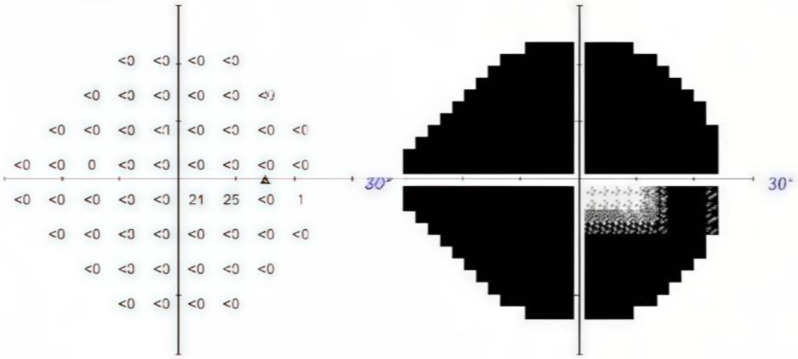
Severe constriction (-30.95 dB) reported on the visual field test

A 66-year-old Chinese female was diagnosed with POAG on the right eye during a long-term follow-up at the outpatient department for several years. The patient had a medical history of bilateral cataract surgery and an underlying disease of diabetes mellitus (DM). The patient regularly visited her ophthalmologist and had been using three types of anti-glaucoma eye drops for more than five years. However, the IOP could not be stably controlled at a baseline of 14 mmHg and progress to 20 mmHg with a cup-to-disc ratio (CDR) of 0.9. Optical coherence tomography (OCT) revealed severe thinning in the retinal nerve fiber layer (RNFL) and ganglion cell complex (GCC), along with a severe constriction (-30.95 dB) on the visual field test (Figs. 1 and 2).

To reduce the IOP, we created a 1.8-mm incision from the temporal side and inserted the HFDS tip, creating six pockets through the TM on the nasal sclera. On postoperative day one, the IOP was 12 mmHg without any glaucoma medications. Fundus examination revealed no changes compared to baseline, with a normal macula, 0.9 CDR, and no evidence of choroidal detachment. Consequently, the patient was prescribed 2% pilocarpine twice per day for four weeks. During the one-month follow-up postoperatively, the IOP remained stable between 11 and 12 mmHg. However, during the follow-up visits at two, three, and four months postoperatively, the IOP was 15, 17, and 19 mmHg, respectively, which was higher than the baseline of 14 mmHg. Therefore, we gradually reintroduced three types of anti-glaucoma eye drops owing to poor IOP control.

During the one-year follow-up after HFDS, no significant structural changes in the RNFL and GCC were observed. The postoperative number of corneal endothelial cells decreased by 13% (1176–1026 cells/mm^2^) in the central area, but no significant change was observed at the nasal side. A B-scan measured an anterior chamber depth, and it was 4.81 mm. The patient continued the same medication regimen, and the IOP remained stable between 13 and 15 mmHg.

### Case 2

**Fig. 3:**
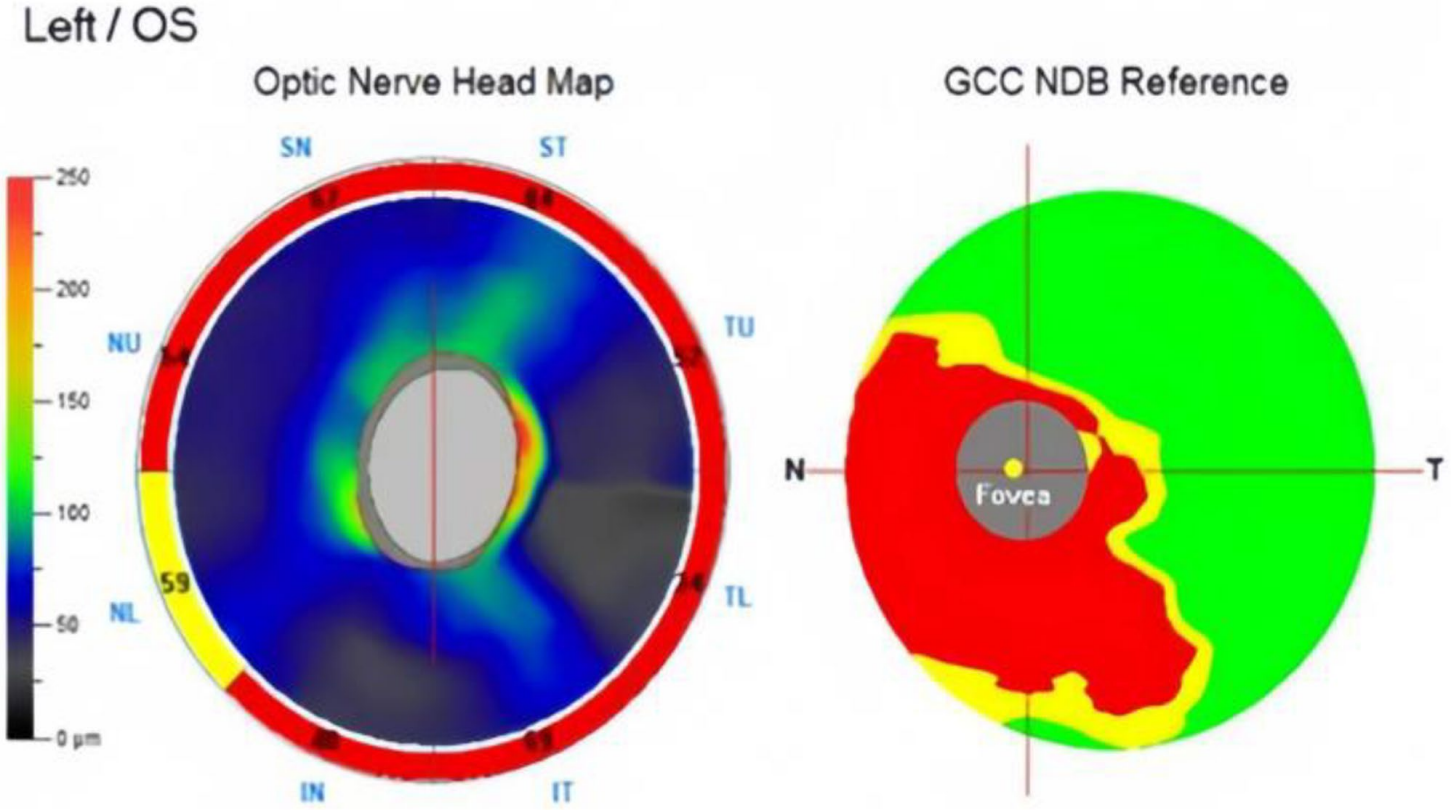
OCT revealed severe thinning in the RNFL and GCC

**Fig. 4:**
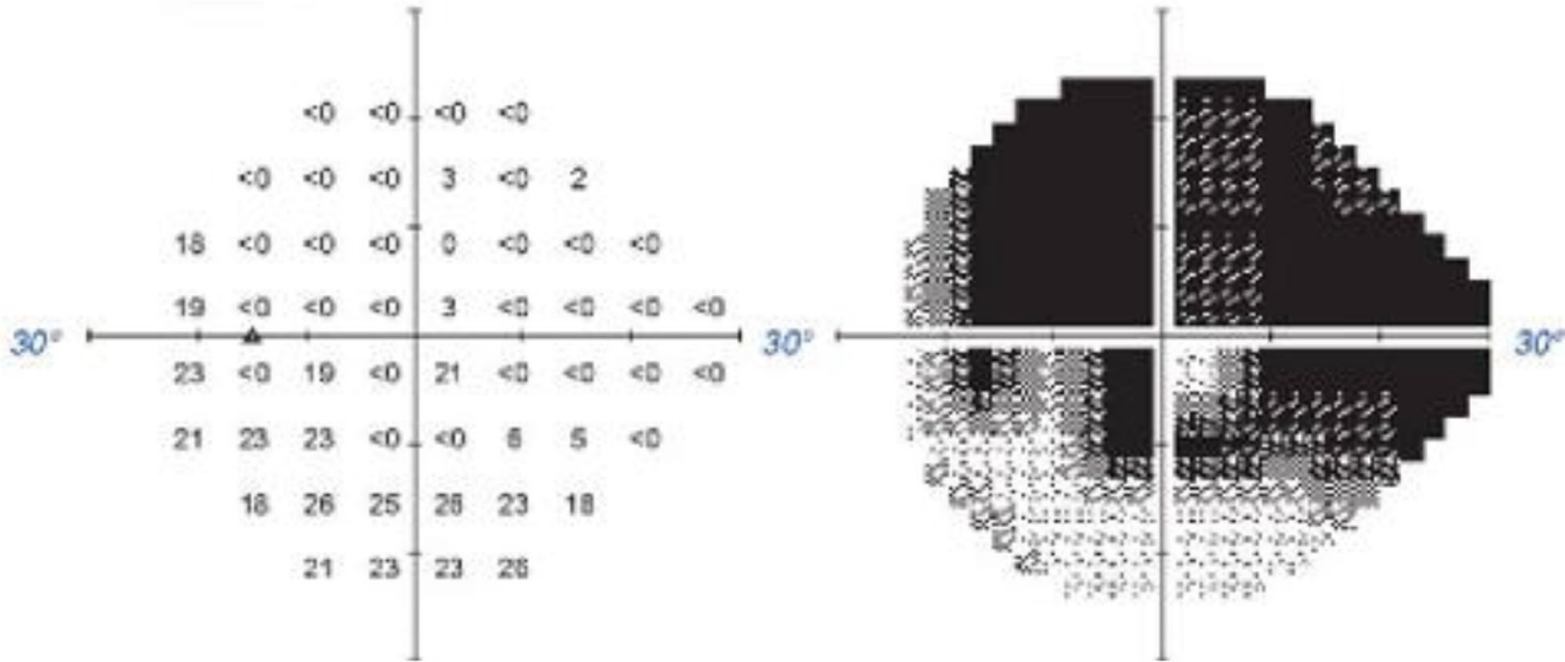
Moderate-to-severe damage (-24.73 dB) reported on the visual field test

A 50-year-old Chinese female was noted to have bilateral POAG during long-term follow-up at the outpatient department for several years. The patient had a medical history of bilateral cataract surgery without any underlying disease. The patient regularly visited her ophthalmologist and had been using three types of anti-glaucoma eye drops for more than six years. However, the patient had blurred vision in the left eye and could not maintain the IOP at a baseline of 13 mmHg for two months. The preoperative IOP of the left eye was 18 mmHg with 0.9 CDR. OCT revealed severe thinning in the RNFL and GCC, along with a moderate-to-severe damage (-24.73 dB) on the visual field test (Figs. 3 and 4).

To reduce the IOP, we created a 1.8-mm incision from the temporal side and inserted the HFDS tip, creating six pockets through the TM on the nasal sclera. On postoperative day one, the IOP was 16 mmHg without any glaucoma medications. Fundus examination revealed no changes compared to baseline, with a normal macula, 0.9 CDR, and no evidence of choroidal detachment. Consequently, the patient was prescribed three types of anti-glaucoma eye drops with 2% pilocarpine twice per day for four weeks. Monthly follow-ups were conducted for six months postoperatively, and the IOP remained stable between 12 and 13 mmHg.

During the one-year follow-up after HFDS, no significant structural changes in the RNFL and GCC were observed. The postoperative number of corneal endothelial cells decreased by 7% (from 2556 to 2363 cells/mm²) at the nasal side, but no significant change was observed on the central area. A B-scan measured an anterior chamber depth, and it was 4.86 mm. The patient continued the same medication regimen, and remained the baseline IOP of 13 mmHg.

## Surgical procedure

Under local ocular anesthesia of proparacaine 0.5% ophthalmic solution, two clear corneal incisions were created 90° apart in the superior and temporal quadrants using a 1.8-mm slit knife. Subsequently, a high-density ophthalmic viscoelastic device (OVD), 0.01% lidocaine, and carbachol were injected into the anterior chamber. The next step involved the application of an OVD to the corneal surface. Following this, the tip of an Abee^®^ glaucoma probe, which is part of the Oertli phacoemulsification machine (Cata-Rhex 3, Switzerland), is carefully inserted through the temporal side port. The probe was then pushed at an opposite angle.

**Fig. 5:**
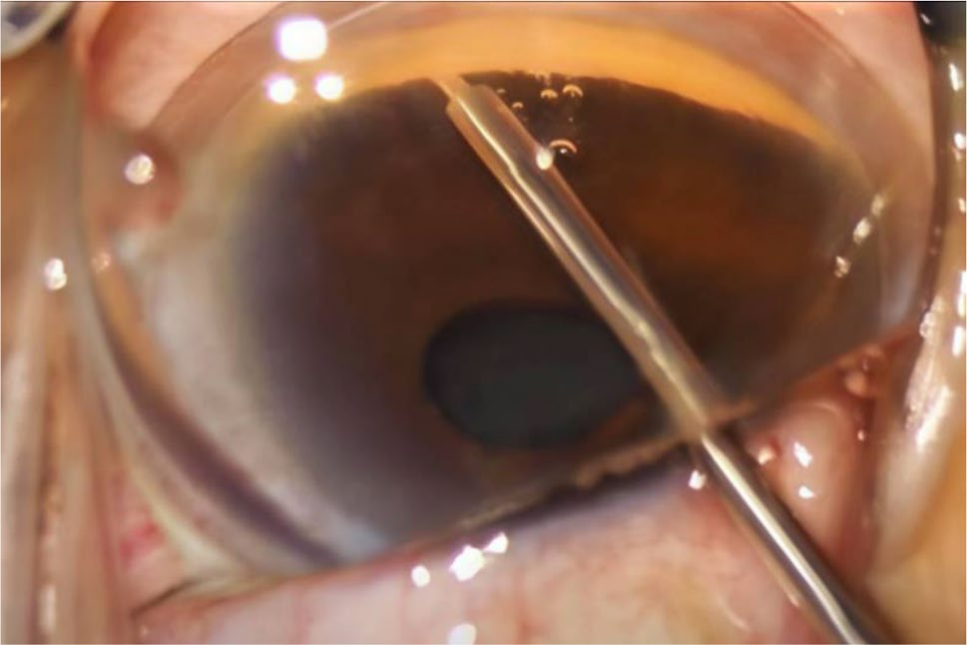
Abee^®^ glaucoma probe tip advanced approximately 1 mm towards the sclera, penetrating through the TM

Once the probe tip passes beyond the edge of the pupil, goniolens (MV LV-50, Phakos, France) can be placed on the cornea to enhance visualization of the anterior chamber angle. By pressing the foot pedal of the Oertli machine, the Abee^®^ glaucoma probe tip is advanced approximately 1 mm towards the sclera, penetrating through the TM until three beeping sounds are heard (Fig. 5). This action was repeated five times to create six pockets on the nasal side of the TM. Each opening is 0.6 mm in width and 0.3 mm in thickness. Subsequently, the OVD was thoroughly washed, and hydration was applied to the side wound to ensure wound closure. Gentamycin and betamethasone ointments were applied and covered with gauze.

## Discussion

HFDS is a minimally invasive glaucoma surgery (MIGS) performed using an internal approach that effectively lowers IOP in patients with glaucoma, particularly in those who do not respond adequately to conventional medical treatments [3]. This report presents two of Taiwan’s first patients with POAG treated by HFDS. The first patient did not use any glaucoma eye drops within the first month after surgery, but returned to the three preoperative types of medication after the fourth month. The second patient continued the original medication postoperatively, and the intraocular pressure remained lower and stable compared to the preoperative levels during the one-year follow-up. Both patients exhibited mild postoperative loss of corneal endothelial cells, a known risk factor associated with various MIGS [2]. However, there were no significant changes in the OCT and visual field test after one year. The stability of the anterior chamber depth postoperatively also indicates the safety of HFDS in maintaining the ocular anatomy.

The electrodes of the probe are placed away from the tissue, and ionization forms an electric arc discharge, generating high-frequency energy dissipation as cutting effect near the electrode tip [4]. Then, the probe creates six pockets in the TM to reduce IOP, enhancing aqueous humor outflow. Compared to other MIGS techniques, HFDS generally showed a more significant initial reduction in IOP. During the one-year follow-up of our study, the IOP in both patients showed a significant reduction compared to preoperative levels, with decreases of 30% and 33.3%, respectively. These results are similar to those of previous studies [2, 5]. El-Shiaty et al. followed 43 patients with open-angle glaucoma (OAG) for nine months after HFDS and reported an average IOP reduction of approximately 39.2% [2]. Pajic et al. followed 53 patients with OAG for 72 months after HFDS and reported an average IOP reduction of approximately 42.5% [5]. In addition to HFDS, the Hydrus Microstent, iStent, and trabectome are other MIGS techniques. Fea et al. used the Hydrus microstent to treat OAG and observed a 26% average reduction in IOP after one year [6]. Hengerer et al. reported that the long-term effects of second-generation iStents in treating OAG resulted in a 42% reduction in IOP [7]. Maeda et al. performed trabectome surgery on 80 patients with OAG and observed a 28.7% average reduction in IOP after six months [8].

HFDS is considered safer than traditional glaucoma surgeries, including drainage tube surgery and trabeculectomy, because of its minimally invasive nature and low incidence of postoperative complications [4]. Traditional surgeries are often associated with significant complications, such as hypotony, hyphema, choroidal detachment, and bleb-related issues. According to Edmunds et al., the complication rate of trabeculectomy was 24% [9], while Gedde et al. reported a complication rate of 13% for drainage tube surgery [10]. In our study, we did not observe any postoperative corneal edema or significant clinical complications. Additionally, endothelial cell loss (ECL) in the two patients was 13% and 7% respectively, one month postoperatively. Compared to previous studies, such as El-Shiaty et al., who reported 4.7% ECL at nine months postoperatively, our results were slightly higher [2]. We speculate that this may be related to the fact that this surgery was performed for the first time and that the first patient had a lower central corneal endothelial cell count of only 1176 cells/mm² preoperatively.

In the first patient with DM, IOP control was inadequate within four months postoperatively; therefore, we gradually reintroduced three types of anti-glaucoma eye drops to maintain a stable IOP. This result could be due to the following three reasons: First, one study reported that inflammatory substances in the anterior chamber, such as advanced glycation end-products in diabetic patients, increase the viscosity of the aqueous humor, impeding its flow and drainage [11]. We speculate that despite using levofloxacin/fluoromethalone eye drops and 2% pilocarpine for one month postoperatively, inflammation in the anterior chamber was not fully controlled, leading to the blockage of surgical incisions by inflammatory materials. Secondarily, another study reported that the trabecular-iris space area in Asians was smaller than in Caucasians [12]. Therefore, we speculate that when using the tip to create a pocket with diathermy, there is a higher likelihood of iris injury, leading to an inflammatory response and even peripheral anterior synechiae (PAS). Third, it is possible that the six pockets created by HFDS have already healed, resulting in poor aqueous humor drainage [13]. HFDS may require more experience and study to prove its efficacy.

## Conclusion

In our experience, HFDS offers several advantages over traditional glaucoma surgeries: it does not affect refractive power; it can be repeated in the same location if necessary; it involves no foreign body, implant, or shunt postoperatively; and it avoids bleb-related filtration complications. Our study showed effective IOP reduction and no severe postoperative complications, highlighting HFDS as a promising option among the MIGS techniques. Future research should focus on optimizing postoperative management, exploring the anatomical differences, and assessing pocket healing to improve HFDS outcomes.

## Data Availability

No datasets were generated or analysed during the current study.
